# Dynamics of B-cell response in MERS-CoV patients and survivors with hybrid immunity

**DOI:** 10.1128/mbio.03356-25

**Published:** 2025-12-16

**Authors:** Hebah A. Al-Khatib, Fatma H. Ali, Hadeel T. Zedan, Maria K. Smatti, Peter V. Coyle, Sara A. Taleb, Ali A. Hssain, Asmaa A. Al-Thani, Hadi M. Yassine

**Affiliations:** 1 QU Health, Qatar University, Biomedical Research Center 61780, Doha, Qatar; 2 Hamad Medical Corporation 36977, Doha, Qatar; 3 Weill Cornell Medicine-Qatar, Qatar Foundation 36578, Doha, Qatar; 4 College of Health Sciences, Qatar University 61780, Doha, Qatar; Johns Hopkins University, Baltimore, Maryland, USA

**Keywords:** MERS-CoV, SARS-CoV-2, B-cell response, cross-reactivity, neutralization, hybrid immunity, vaccination

## Abstract

Middle East respiratory syndrome coronavirus (MERS-CoV) causes a highly lethal respiratory infection for which no vaccines or antiviral therapeutics are currently available. Understanding the immune response is critical for designing effective therapeutics. Here, we comprehensively characterized the dynamics of B-cell responses in severely infected MERS-CoV patients and survivors with SARS-CoV-2 exposure history. Infected patients developed robust neutralizing antibody responses within 1 month of illness, with moderate-to-high cross-neutralization activity against SARS-CoV-2. The enhanced neutralization activity coincided with an increased abundance of specific mutated, class-switched IgG clones. Notably, one such clone was detected at moderate prevalence in both patients, and its expansion was accompanied by high neutralization activity against both viruses. Conversely, MERS-CoV survivors demonstrated higher neutralization activity against MERS-CoV after vaccination, despite minimal changes in antibody titers and limited alterations in B-cell repertoire properties. This suggests that the enhanced neutralization activity may be mediated by the reactivation and expansion of cross-reactive memory B cells targeting conserved epitopes, originally generated in response to the virus that triggered the primary immune response. These findings provide valuable insights into the B-cell repertoire landscape during natural MERS-CoV infection and highlight the potential for identifying broadly neutralizing antibodies in individuals with hybrid immunity.

**IMPORTANCE:**

This study examines the immune responses of MERS-CoV patients and survivors who have had confirmed exposure to SARS-CoV-2. It offers a unique opportunity to characterize cross-reactive B-cell responses in individuals possessing hybrid immunity to both pathogenic coronaviruses. To our knowledge, no previous studies have examined longitudinal changes in the B-cell repertoire in MERS-CoV patients or survivors before and after SARS-CoV-2 vaccination. Our findings reveal enhanced neutralization activity against both MERS-CoV and SARS-CoV-2 following infection or vaccination, which appears to be associated with distinct patterns of B-cell repertoire dynamics. Notably, the data strongly suggest the presence of potent cross-neutralizing antibody responses, particularly in MERS-CoV patients, driven by dominant B-cell clones. These results underscore the potential for identifying broadly neutralizing antibodies in individuals with hybrid immunity.

## INTRODUCTION

Middle East respiratory syndrome coronavirus (MERS-CoV) has caused 2,605 cases and 936 deaths since its emergence in 2012 (36% mortality rate) (1, 2). Transmission primarily occurs through direct or indirect contact with dromedary camels, although limited human-to-human transmission has also been documented in healthcare settings and among household members (3, 4). MERS-CoV circulates at high prevalence in camels and continues to cause severe, sporadic outbreaks in humans, raising concerns about global preparedness for future outbreaks.

The clinical presentation of MERS-CoV infection ranges from mild or asymptomatic cases to severe respiratory failure and death (5, 6). Antibody responses typically develop 14–21 days after symptom onset (7). Antibody persistence correlates with disease severity, with severe cases maintaining higher neutralizing titers for over a year compared to lower, short-lived responses in mild infections (8). The inefficient immune response and high mortality rate among hospitalized patients highlight the urgent need for effective preventative and therapeutic interventions.

Despite significant advances in MERS-CoV research, no vaccines or therapeutic antibodies have been approved to prevent or treat MERS-CoV infections. Current vaccine and antibody candidates primarily target the surface spike (S) protein that mediates the virus binding, fusion, and entry into host cells (9). The spike protein binds its respective host cell receptor through the receptor-binding domain (RBD) located in the S1 subunit (10). Several promising monoclonal antibodies have been identified, most of which target the RBD, while a few bind the S2 subunit and the N-terminal domain of the S1 subunit (9, 11, 12). These antibodies have been derived from different sources, including human antibody libraries, transgenic humanized mice, transchromosomic bovines, camel nanobodies, and convalescent individuals (13–16). To date, however, no antibodies have been isolated from individuals with natural hybrid immunity, likely due to the extreme rarity of individuals who survived MERS-CoV infections.

This project takes advantage of a rare cohort of individuals with hybrid MERS-CoV/SARS-CoV-2 immunity, providing a unique opportunity to characterize antibody response in those patients. Cross-neutralizing activity has been observed in MERS-CoV survivors following SARS-CoV-2 infection or vaccination (17, 18). Therefore, a deep understanding of cellular and humoral immunity in those patients could significantly advance therapeutic strategies against MERS-CoV and pathogenic coronaviruses. In this study, we explored the dynamics and properties of antibodies and the B-cell receptor (BCR) repertoire in MERS-CoV patients and survivors with prior exposure to SARS-CoV-2.

## MATERIALS AND METHODS

### Study groups

**Fig 1:**
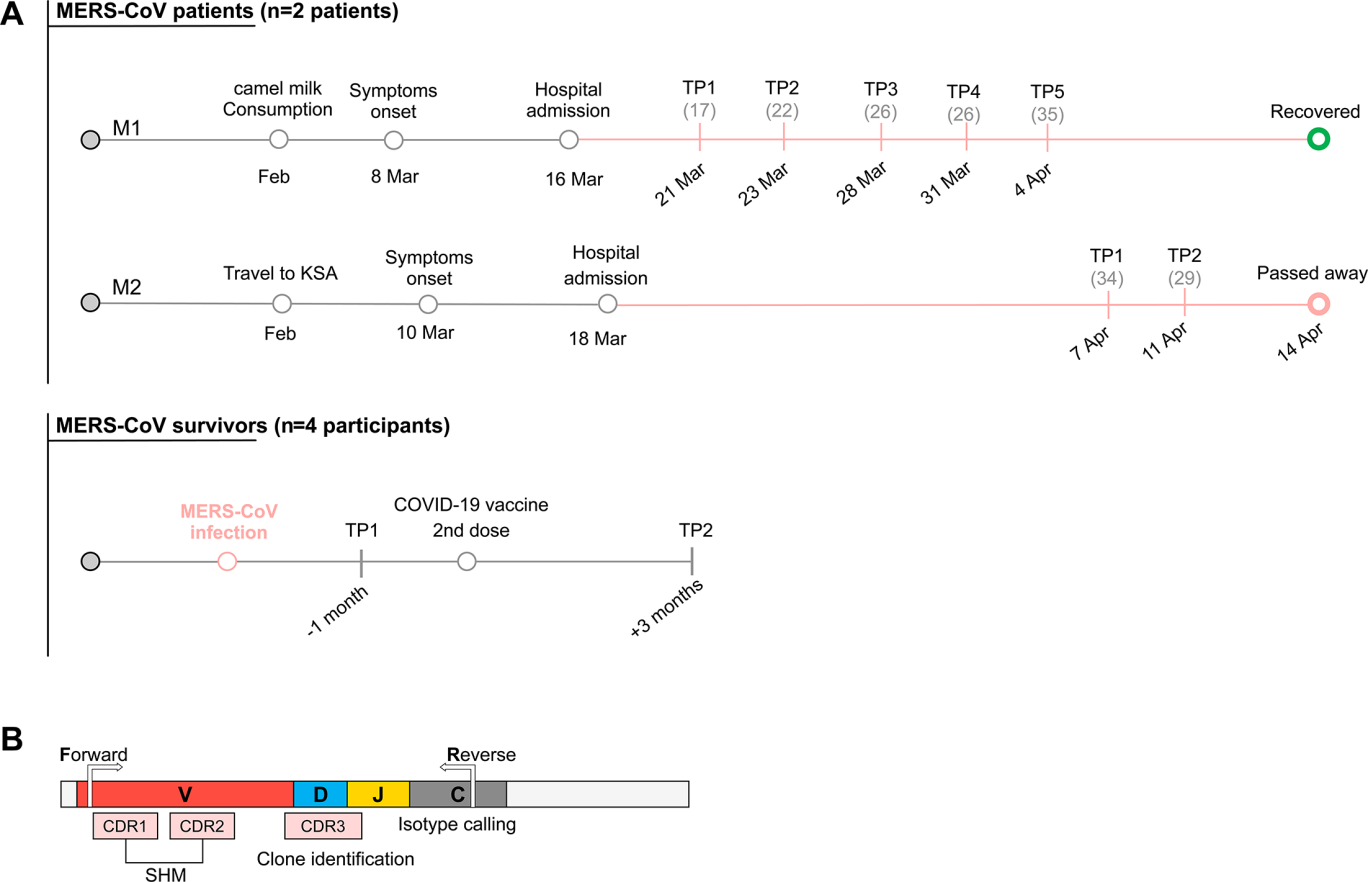
Schematic representation of sample collection timeline and B-cell receptor (BCR) sequencing strategy. (**A**) Schematic representation of sample collection dates from infected MERS-CoV patients (group I) and vaccinated MERS-CoV survivors (group II). The timeline includes travel history, hospital admission dates, and Ct values (shown as gray numbers in brackets) from real-time PCR targeting the upE and orf1a genes of SARS-CoV-2. “TP” indicates time points on the graph. (**B**) Schematic overview of BCR sequencing coverage. CDR1 and CDR2 regions were used to estimate the frequency of somatic hypermutation (SHM); CDR3 was used for clone assignment; and the C region was used to identify clone isotypes.

Blood samples were collected from two MERS-CoV-infected patients (group I [GI]) and four MERS-CoV survivors (group II [GII]). Serial blood samples were collected from group I patients during a month of infection, while blood samples from group II individuals were collected before and after receiving the second dose of SARS-CoV-2 mRNA vaccine (Fig. 1A; Table S1). A detailed demographic and clinical description of the two MERS-CoV cases is published on the WHO website (19). Briefly, both cases were reported in Qatar in 2022. Both cases had frequent close contact with dromedary camels and consumption of camel’s milk. The first patient (M1, 50-year-old male) presented to the emergency department with cough, fever, and shortness of breath. The second patient (M2, 85-year-old male) was admitted to the emergency department with symptoms similar to those presented in the first case. On 22 March, the patient was transferred to the ICU, where he stayed for 24 days before passing away on 14 April. MERS-CoV infection was confirmed in both patients by RT-PCR targeting upE and orf1a genes. Six blood samples were collected from the first patient and two from the second patient.

### Processing of blood samples

Whole blood samples were collected into EDTA tubes. Sera were separated, aliquoted, and stored at −80°C. PBMCs were isolated using the Ficoll-Paque Plus reagent (Cytiva, USA) as per the manufacturer’s protocol. In brief, a whole blood sample was diluted and layered on top of Ficoll-Paque reagent. This was followed by a centrifugation step at 400 × *g* for 30–40 minutes at room temperature. The buffy coat layer was aspirated and rinsed twice with 1× phosphate-buffered saline (PBS). Cells were resuspended in complete RPMI-1640 medium, counted, and subjected to a final round of centrifugation. Cell pellets were then resuspended in fetal bovine serum containing 10% DMSO and stored in the liquid nitrogen (5 × 10^6^ cells per vial).

### Serological analysis

#### ELISA

Titers of anti-MERS-CoV and anti-SARS-CoV-2 antibodies were measured by enzyme-linked immunosorbent assay (ELISA) using full-length spike, S1-RBD, and S2 proteins (SinoBiological, USA). Proteins were coated onto ELISA plates (Thermo Fisher Scientific, USA) for 24 hours. Plates were washed and incubated with blocking buffer containing 1% bovine serum albumin for 1 hour at 37°C. Plates were washed with PBS containing 0.1% Tween-20. Serially diluted samples were then added to the coated wells and incubated for 1 hour at 37°C. Horseradish peroxidase-labeled polyclonal rabbit antibody targeting human IgG, IgM, and IgA (Abcam, USA) was added to each well and incubated for an additional 1 hour. KPL SureBlue TMB Microwell Peroxidase Substrate was then added and incubated for 5–10 minutes (Sera Care, USA). The plates were read using the BioTek Cytation 5 Microplate Reader (Agilent, USA). Antibody titers were calculated using the endpoint analysis, and cut-off values were determined to be three times the average of the blank reading. All experiments were conducted in triplicate.

#### Pseudovirus neutralization assay

The neutralization activity was measured using MERS-CoV and SARS-CoV-2 (Wuhan-hu-1) pseudoviruses (PVs) generated by VSV pseudotyping systems as previously described (20, 21). Cells and plasmids were kindly provided by Viral Pathogenesis Laboratory, Vaccine Research Center, National Institutes of Health. In brief, Huh7.5 and HEK293T-ACE2 cells—for MERS-CoV and SARS-CoV-2, respectively—were seeded in a 96-well white/black isoplate (PerkinElmer, USA). Heat-inactivated serum samples were diluted (twofold dilution) and incubated with the pseudovirus for 2 hours at 37°C, then added to the cells. Twenty-four hours later, cells were lysed, and 50 µL of Luciferase substrate was added to each well to measure PV entry (Bio-Glo Luciferase Assay System; Promega, USA). Luciferase activity was measured using a luminescence plate reader (Tecan Infinite 200 PRO), and the percentage of inhibition was calculated for each sample.

### B-cell repertoire analysis

#### Sequencing of BCR

Total RNA was extracted from 5 × 10^6^ cells using the RNeasy Mini kit (Qiagen, USA) according to the manufacturer’s instructions. The quality of extracted RNA was evaluated using Agilent Bioanalyzer 2100. Amplification and sequencing of the heavy chains of B-cell receptors were performed using the iRepertoire iR-Complete Dual Index Primer Kit (iRepertoire, USA), as described in the manufacturer’s protocol (Fig. 1B). In brief, reverse transcription was conducted using 400 ng of RNA, followed by dual-index PCR for sample barcoding. PCR products were purified, quality-checked with the High-Sensitivity DNA Kit (Agilent), and pooled in equimolar amounts (150 ng per sample). Libraries were quantified (JetSeq Lo-ROX kit), spiked with 10%–15% PhiX, and sequenced as paired-end reads (250 × 250 bp) using the MiSeq kit (v.2). A negative control was included in the run to ensure a contamination-free library preparation process. All samples were sequenced in duplicates. Unfortunately, the number of sequences obtained from time point 4 in M1 was not enough to conduct the BCR analysis.

#### Processing of B-cell receptor sequences

Raw sequencing reads were processed using the iRweb analysis pipeline. Pre-processing sequencing reads included trimming low-quality ends and stitching the paired-end reads. Stitched reads, with an average overlapping region of 120 bp, that are not 100% identical within the overlap region were discarded. Reads were then collapsed based on nucleotide sequence identity. The stitched reads (~500 bp) were then submitted to IMGT/HighV-QUEST for V(D)J germline assignment, immunoglobulin gene use, and sequence annotation. Output results were filtered to remove non-productive reads using the Change-O toolkit (v.1.3.0) (22). Novel V genotypes were called using the TIgGER R package and used to correct V allele calls generated by the IMGT database. Samples collected at different time points from the same patient were clustered together to allow easy comparison of clusters over time. Clonotyping was performed using the Change-O toolkit by applying the cut-off value determined in the Shazam R package (v.1.1.2). Shazam R package was used to estimate the optimal distance threshold that separates clonally related from unrelated sequences using single nucleotide Hamming distance model (22). Clonotype size was inferred from the number of sequences in each unique clonotype. Clonotypes containing only one read were considered to result from sequencing bias and were removed before the subsequent analysis (except for diversity analysis).

#### Clonal diversity

B-cell repertoire diversity was estimated using VDJtools (23). Diversity was estimated using the normalized data sets by resampling to the size of the smallest data set. The diversity of normalized data sets was presented as Shannon-Wiener index (diversity) and inverse Simpson index (dominance) to measure the number of unique clones and their abundance in each sample. Clonal diversity was also estimated using the D50 index as a quantitative measure of clonal expansion.

#### Somatic hypermutations and clone convergence

Somatic hypermutation (SHM) frequency—including replacement and silent mutations—per unique VDJ region per time point and isotype were calculated using the CDR region for each sample using the observedMutation function within the SHazaM package (22). Convergent clones were identified based on shared V and J gene segments, identical CDR3 length, and a minimum of 85% amino acid sequence identity within the CDR3 region.

### Statistical analysis

Correlation analyses were conducted and visualized using the Spearman correlation test in Prism 10. Statistical significance was assessed using non-parametric tests, including the Wilcoxon test, Kruskal-Wallis test, or Mann-Whitney test, as appropriate.

## RESULTS

### Virus-specific antibody titers correlate with neutralization activity

**Fig 2:**
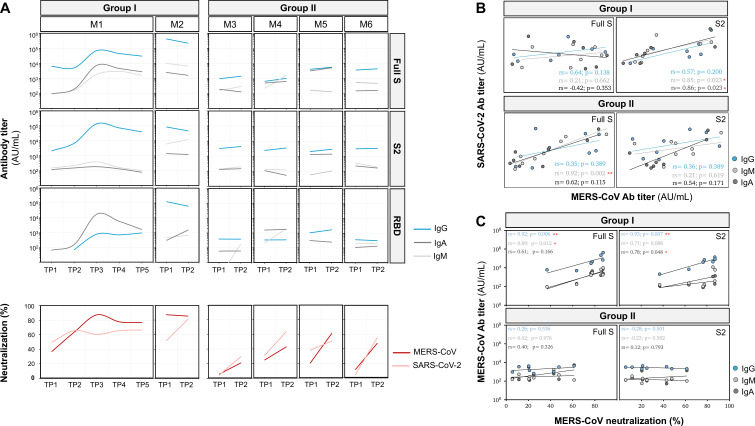
Serology profiles of infected and vaccinated groups at different time points. (**A**) Antibody titers against MERS-CoV spike protein: full-length spike (S) protein, receptor-binding domain, and S2 subunit. The panel shows changes in antibody titers during acute infection (group I) and before and after the SARS-2 vaccination (group II). The lower panel (in red) displays neutralization levels against MERS-CoV and SARS-CoV-2 pseudoviruses. (**B**) Correlation between anti-MERS-CoV and anti-SARS-CoV-2 titers. The panel shows Spearman correlation coefficients (*r_s_*) between MERS-CoV and SARS-CoV-2 antibodies targeting the S and S2 subunits. (**C**) Correlation between MERS-CoV antibody titers and neutralization levels. Plots display the relationship between MERS-CoV neutralization levels and titers of antibodies targeting MERS-CoV full S and S2 proteins. Each point represents a time point. Spearman correlation coefficient *r*_*s*_ and *P* values are shown for each comparison. Red asterisks denote statistical significance: *P < 0.05, **P < 0.01, ***P < 0.001.

Analysis of MERS-CoV antibody titers in both groups was quantified using the full-length S, RBD, and S2 subunit. In GI patients, anti-MERS-CoV antibodies targeting all three protein domains were detected at all time points. The first case (M1) exhibited peak antibody titers against all proteins 20 days post-symptom onset, which remained elevated for the subsequent 10 days. Similarly, the second case (M2) maintained high and stable antibody titers 1 month after symptom onset. Notably, the anti-S IgG response in M1 was predominantly driven by anti-S2 antibodies, while M2 showed a combined anti-RBD and anti-S2 response. In contrast, GII participants showed modest changes in anti-MERS-CoV antibody titers following SARS-CoV-2 vaccination, regardless of antibody isotype (Fig. 2A).

Given the reported cross-reactivity among betacoronaviruses, we also assessed anti-SARS-CoV-2 antibody levels in both groups (Fig. S1A). GI patients exhibited detectable anti-SARS-CoV-2 spike antibodies at all time points, albeit at lower levels compared to MERS-CoV antibodies. Anti-RBD antibody levels remained stable over time, irrespective of isotype. Interestingly, anti-S2 IgG antibodies showed a gradual increase, peaking around 20 days post-infection, mirroring the pattern observed for anti-MERS-CoV IgG antibodies. The anti-SARS-CoV-2 antibody response was primarily driven by anti-S2 antibodies, with a comparatively weak anti-RBD response observed in both cases. Correlation analysis of MERS-CoV and SARS-CoV-2 antibody titers in GI patients revealed a positive correlation for both IgM (rs = 0.85, *P* = 0.023) and IgA (rs = 0.86, *P* = 0.023) antibodies targeting the S2 regions of both viruses (Fig. 2B).

As anticipated, GII participants demonstrated increased anti-SARS-CoV-2 antibody titers following vaccination. This response was primarily mediated by IgG and, to a lesser extent, IgA antibodies, predominantly targeting the S2 protein (Fig. S1A). However, the elevated anti-S2 antibody titers in the GII group did not show a significant correlation with anti-S2 titers against MERS-CoV (Fig. 2B). The enhanced antibody response against the SARS-CoV-2 S2 protein can be attributed to the amino acid sequence homology in the S2 subunit between MERS-CoV and SARS-CoV-2.

We further assessed the antibody function using the pseudovirus neutralization assay. Particularly, we explored the neutralization capacity of antibodies against MERS-CoV and SARS-CoV-2 pseudoviruses (Fig. 2A). In GI patients, the neutralization activity against MERS-CoV pseudovirus mirrored the antibody titer patterns observed in ELISA. The neutralization activity increased gradually and reached its highest level after 20 days of symptom onset in M1 (87%). The neutralization activity decreased slightly at later time points but remained relatively high (more than 75%). Similarly, M2 demonstrated a remarkably high MERS-CoV neutralization activity after a month of symptom onset. Group I patients have also shown a moderate-to-high neutralization activity against SARS-CoV-2 pseudovirus at all time points. In M1, SARS-CoV-2 neutralization activity was comparable to MERS-CoV in the first two time points and stabilized at 65% in later samples. M2 exhibited a dramatic increase in SARS-CoV-2 neutralization activity from 51% at the first time point to 81% at the second time point. In GII participants, neutralization activity increased two- to fivefold against both viruses following SARS-CoV-2 vaccination. This increase was comparable for both pseudoviruses, with MERS-CoV neutralization reaching 61% and 48% in M5 and M6 samples, respectively (Fig. 2A).

To evaluate the relative quality of antibodies induced by MERS-CoV infection and SARS-CoV-2 vaccination, we analyzed the relationship between antibody titers and neutralization activity. Significant positive correlations were observed between MERS-CoV IgG titers and MERS-CoV neutralization activity in GI patients (full S: *r*_*s*_ = 0.92, *P* = 0.006; S2: *r*_*s*_ = 0.93, *P* = 0.007) (Fig. 2C), and between SARS-CoV-2 IgG titers and SARS-CoV-2 neutralization levels in GII participants (full S: *r*_*s*_ = 0.88, *P* = 0.007; S2: *r*_*s*_ = 0.81, *P* = 0.021) (Fig. S1B). This pattern suggests that antibody titers may serve as indicators of neutralizing capacity, but the relationship appears to be specific to the primary antigenic stimulus.

Finally, we performed a correlation analysis between isotype usage derived from repertoire analysis and serum immunoglobulin titers obtained from ELISA. Overall, no significant correlations were seen between isotype usage and antibody titers in both groups. A weak positive correlation was seen between proportions of IgM and IgA B cells and corresponding anti-S and anti-S2 antibody titers in GII participants. The lack of correlation between BCR repertoire and serum titers, particularly in infected patients (GI), is likely to be due to differences in cellular and immunoglobulin kinetics. Following infection, IgG takes time to build up in serum, lagging behind the cellular response. Together, these findings indicate that the correlation between serum Ig and BCR repertoire may not occur, particularly in the setting of acute disease (Fig. S2).

### Reduced clonal diversity in infected patients compared to vaccinated survivors

**Fig 3:**
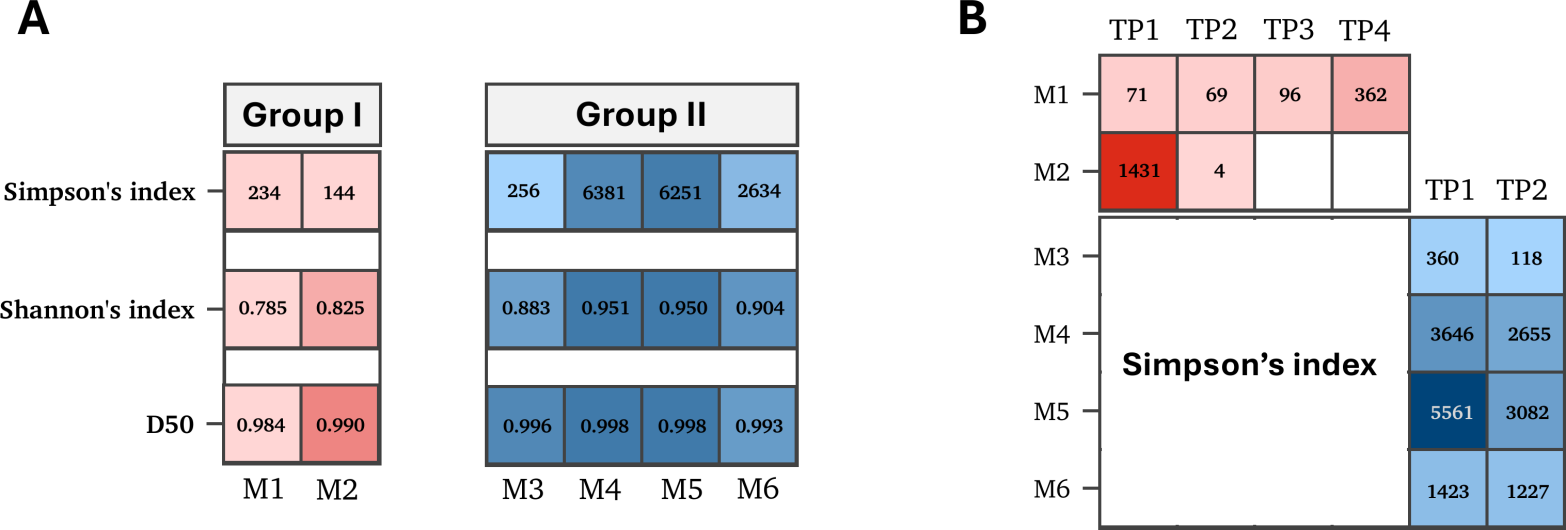
Diversity of B-cell repertoire in infected and vaccinated individuals. (**A**) Heatmap of B-cell repertoire diversity for each individual. Color intensity represents mean values of Simpson’s index, Shannon’s index, and D50 for each individual. (**B**) Heatmap showing temporal changes in B-cell diversity in each individual. Columns represent time points. For infected individuals, time points are days post-symptom onset. For vaccinated individuals, time points are pre-vaccination and post-vaccination.

Assessment of the clonal diversity was performed after subsampling to correct for differences in sequencing depth (Fig. 3). Diversity was assessed using Shannon index (richness and evenness), Simpson’s index (clonal dominance), and D50 index (dominant clone contribution). Overall, vaccinated individuals (GII) exhibited higher B-cell diversity compared to infected patients (GI) regardless of sample collection time (Fig. 3A). Simpson’s index was then used to assess clonal expansion during infection (GI) or following vaccination (GII) due to its high sensitivity to clonal abundance. Analysis of B-cell diversity in GI patients revealed a relatively low B-cell diversity in M1 during the first 20 days of infection, which was accompanied by increased antibody titers and neutralization activity. Similar to M1, M2 demonstrated high diversity after a month of symptom onset (Fig. 3B). Vaccinated participants (GII) also exhibited a modest decrease in B-cell diversity following vaccination (Fig. 3B). This reduction was accompanied by enhanced neutralization activity against both MERS-CoV and SARS-CoV-2.

### Dynamic B-cell isotype switching during MERS-CoV infection

**Fig 4:**
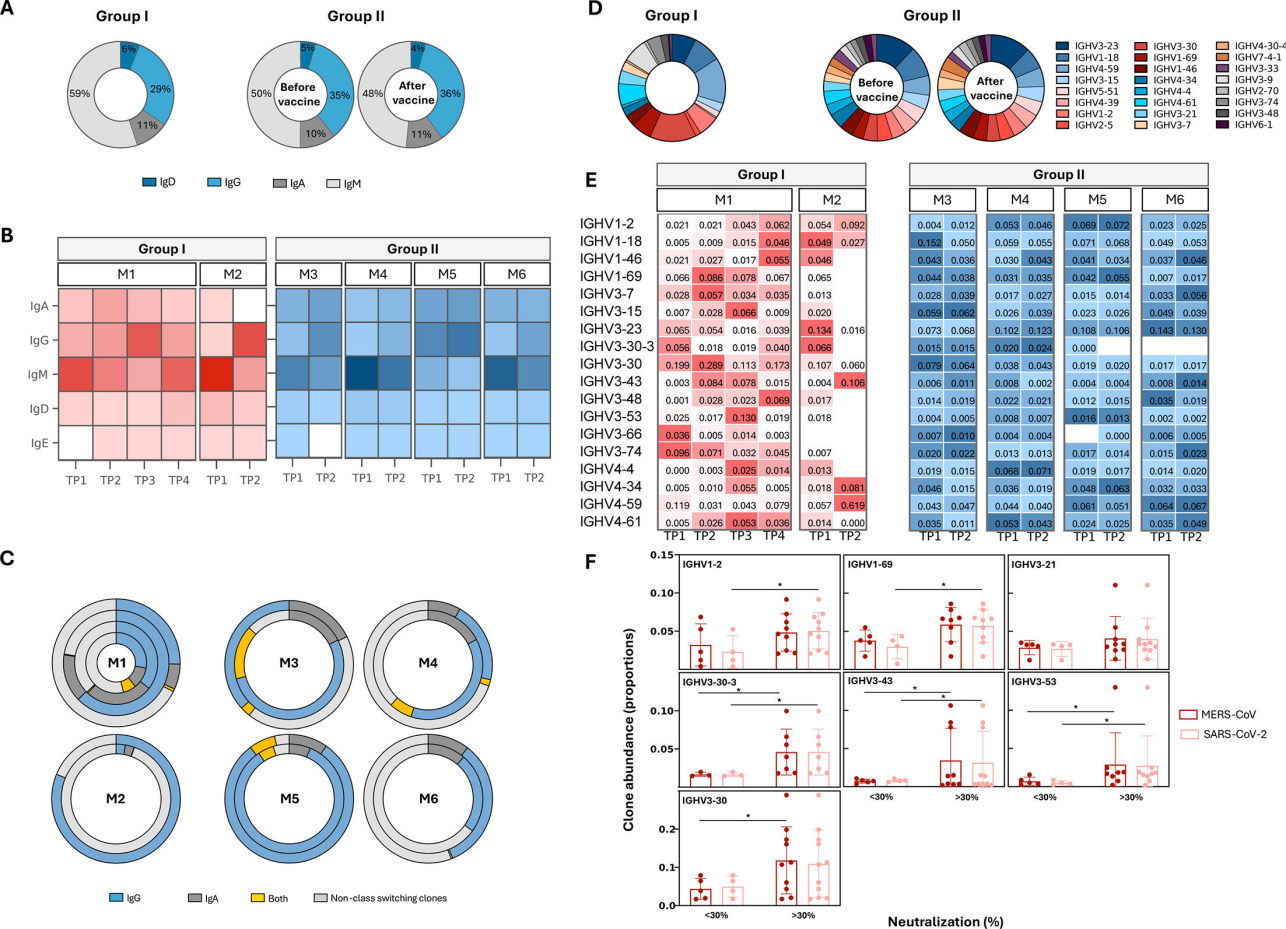
Temporal dynamics of B-cell clones in MERS-CoV-infected patients (group I) and vaccinated MERS-CoV survivors (group II). (**A**) Bar chart illustrating the average abundance of B-cell isotypes in both groups. (**B**) Heatmap depicting changes in the mean abundance of B-cell isotypes for each individual over time. Color intensity indicates the mean number of clones using a specific C gene. (**C**) Circular plots demonstrating changes in the mean number of class-switching clones in individual subjects. Each concentric circle represents a distinct time point, with the innermost circle showing the earliest time point and the outermost circle representing the latest time point. Different colors indicate various antibody isotypes (e.g., IgM, IgG, and IgA). The size of each segment within a circle corresponds to the relative abundance of clones for that particular isotype at the given time point. (**D**) Pie charts illustrating the average V gene usage in both groups. (**E**) Heatmap depicting changes in abundance of clones utilizing different V genes over time in each individual. Only the top 18 most frequently used V genes are displayed. Color intensity corresponds to the mean abundance of clones using a specific V gene. (**F**) Bar plot comparing the abundance of certain clones between samples with weak (<30%) and moderate-to-high (>30%) neutralizing activity. The *Y*-axis represents the relative abundance of clones utilizing specific IGHV genes.

To investigate the kinetics of B-cell responses during MERS-CoV infection and following SARS-CoV-2 vaccination, we examined the proportions of B-cell isotypes at various time points (Fig. 4). Analysis of B-cell isotypes proportions was performed by counting each unique CDR3 sequence once to avoid bias from clonal expansion. Overall, no significant differences were observed between groups, nor were there notable changes in GII following vaccination (Fig. 4A). The longitudinal analysis in GI revealed dynamic shifts, particularly in IgG and IgM proportions (Fig. 4B). The proportions of these two isotypes showed an inverse trend over time, consistent with typical infection-associated class switching, where IgM levels decrease as IgG levels increase. The rise in IgG clones (3-fold increase at TP3 in M1 and 30-fold increase at TP2 in M2) coincided with increased antibody titers and enhanced neutralization against both viruses. IgA clones were detected at early time points but declined thereafter and were undetectable after 1 month in both patients. In contrast, GII showed less pronounced changes. As expected, IgG clone proportions increased post-vaccination (1.9-fold increase), accompanied by a modest rise in IgA and a decline in IgM.

Proportions of isotype-switching clones increased over time in GI. In M1, switching to IgA dominated at TP2 (68%), while IgG dominated at TP3 (68%). In M2, class-switched clones increased to 82% after a month primarily to IgG. In GII, vaccination led to a predominance of IgG-switched clones (Fig. 4C).

### Distinct V gene usage and clonal expansion patterns in GI and GII

To identify potentially enriched V genes, the relative frequencies (number of clones) and clonal abundance (clone size) were analyzed. Clones were derived from 38 unique V genes in GI patients and 55 in GII participants. The most frequently utilized genes in both groups were IGHV3-30, IGHV4-59, IGHV1-18, and IGHV3-23 (Fig. 4D).

Longitudinal analysis of GI patients revealed dynamic changes in clonal abundance during infection. Both patients demonstrated expansion of IGHV3-43 and IGHV4-34 clones at time points corresponding to peak neutralization activity against both viruses. In M1, increased neutralization also coincided with increased abundance of IGHV1-69, IGHV3-7, IGHV3-15, IGHV3-30, IGHV3-53, IGHV4-4, and IGHV4-61 clones. In M2, the expansion of IGHV4-59 clones was particularly associated with stronger SARS-CoV-2 neutralization. In contrast, no major changes in V gene usage were observed in GII following vaccination. Moreover, longitudinal analysis of GII participants did not reveal uniform changes in clonal abundance following vaccination. Instead, neutralization activity in GII participants was linked to the expansion of distinct B-cell clones, including IGHV1-46 in M4 and M6, IGHV4-34 in M5, and IGHV3-43/IGHV3-7 in M6 (Fig. 4E).

In both groups, samples with higher neutralization activity (>30%) had significantly increased clonal abundance of IGHV3-30-3 (*P* = 0.04 for both viruses), IGHV3-53 (*P* = 0.03 for SARS-CoV-2 and *P* = 0.032 for MERS-CoV), and IGHV3-43 (*P* = 0.04 for SARS-CoV-2 and *P* = 0.01 for MERS-CoV) clones (Fig. 4F).

### Low levels of somatic hypermutations in most expanded clones

**Fig 5:**
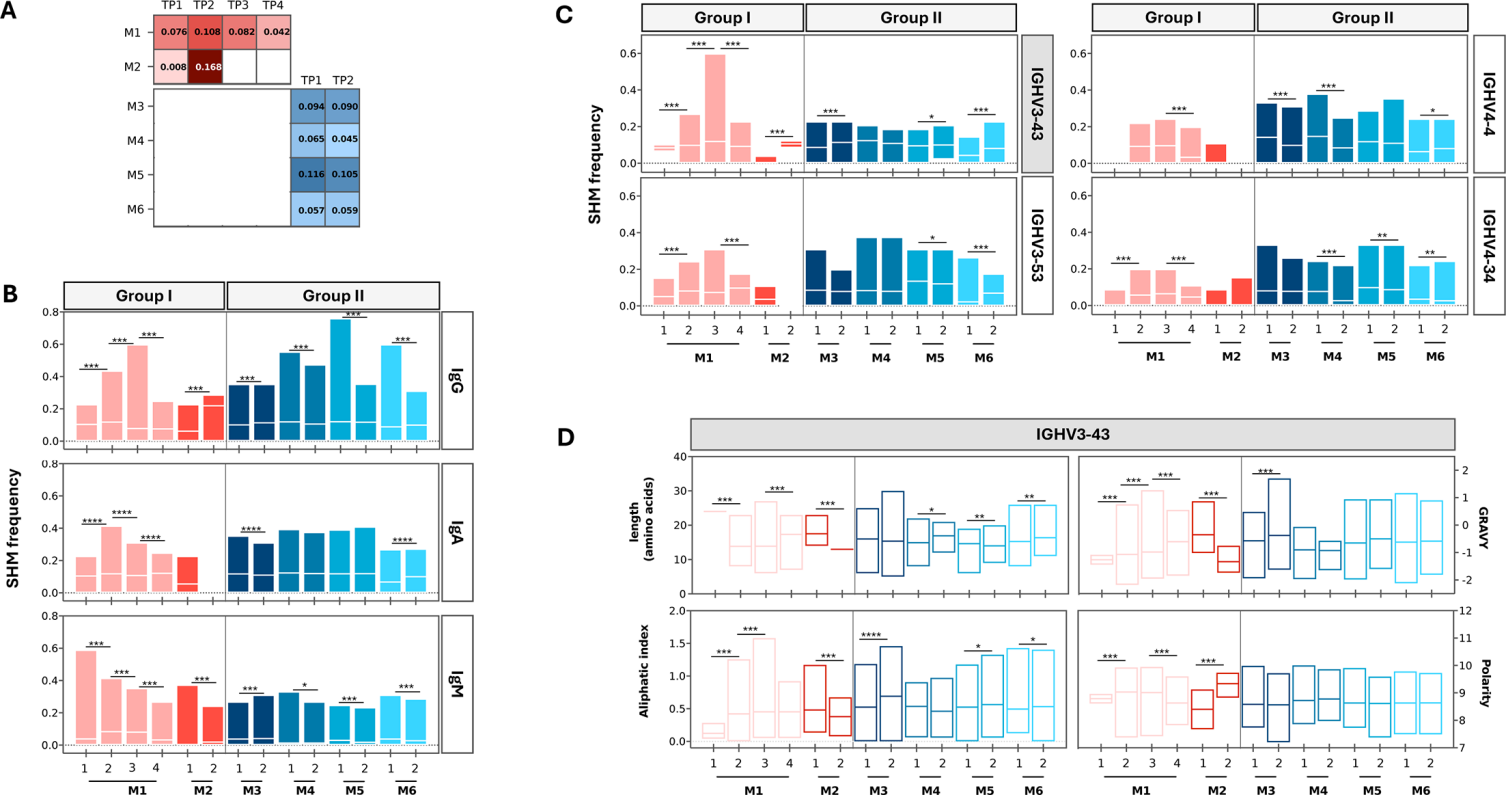
Landscape of somatic hypermutation (SHM) frequency in different B-cell isotypes. (**A**) Average frequency of replacement mutations in each individual. (**B**) Temporal changes in the mean frequency of SHM in IgG, IgM, and IgA B-cell isotypes. (**C**) Bar charts demonstrating changes in SHM frequency in highly mutated B-cell clones. (**D**) Changes in physicochemical properties of the IGHV3-43 clone.

To assess B-cell maturation, the frequency of replacement SHM in the CDR regions was analyzed (Fig. 5). Overall, there were no significant differences in SHM frequencies between GI (mean = 0.081, SD = 0.0503) and GII (mean = 0.079, SD = 0.024). Moreover, no substantial changes in SHM frequency were observed in GII participants following vaccination. In contrast, longitudinal analysis of GI patients revealed dynamic changes in SHM frequency over time. In M1, the highest SHM frequency was seen after 15 days of symptoms (mean = 10.8%, SD = 0.06). The reduced SHM frequency at later time points suggests active selection within germinal centers. In M2, a 10-fold increase in SHM frequency was seen after a month of symptom onset. Interestingly, the rise in SHM frequency in GI coincided with enhanced neutralization activity against MERS-CoV in M1 and SARS-CoV-2 in M2 (Fig. 5A).

Analysis of SHM at the isotype level revealed dynamic changes in SHM frequency in IgG and IgM clones, while IgA clones maintained relatively stable levels. Among all isotypes, IgG clones exhibited the highest SHM frequencies in both groups (mean = 11%, SD = 5.6%) (Fig. 5B). In GI, the increase in IgG SHM observed after 15 and 26 days of infection in M1 and M2 (*P* < 0.001), respectively, compared to previous time points, was followed by a significant increase in neutralization activity. Similarly, the increased SHM in IgG clones after vaccination (*P* < 0.001) in M3, M5, and M6 participants coincided with stronger neutralization activity.

Analyzing SHM in IgG clones utilizing different V genes revealed weak correlations between MERS-CoV neutralization and SHM frequency in most abundant clones, including IGHV3-23, IGHV1-18, IGHV3-30, and IGHV3-21. In contrast, less abundant clones, IGHV3-43, IGHV3-53, IGHV4-4, and IGHV4-34, exhibited increased mutation frequencies at time points corresponding to enhanced neutralization against both viruses in both groups (Fig. 5C). The increased SHM in IGHV3-43 clones, particularly, coincided with higher neutralization activity against both viruses in both groups. Physicochemical analysis of IGHV3-43 clones showed changes indicative of improved binding affinity, including reduced CDR3 length and increased polarity and aliphatic index at time points corresponding to enhanced neutralization (Fig. 5D). In contrast, the increased SHM frequency in several IgG clones coincided with higher SARS-CoV-2 neutralization in GI (Fig. S3A). In M2, the increased SHM frequency in IGHV3-43 and IGHV4-59 IgG clones (10- and 25-fold increases relative to TP1, respectively) coincided with the significant rise in SARS-CoV-2 neutralization. These changes were accompanied by a marked decrease in CDR3 length and increased polarity, suggesting that SHM contributed to increased binding affinity and antigen specificity (Fig. 5D; Fig. S3B).

### Clone convergence between MERS-CoV patients is present but rare

**Fig 6:**
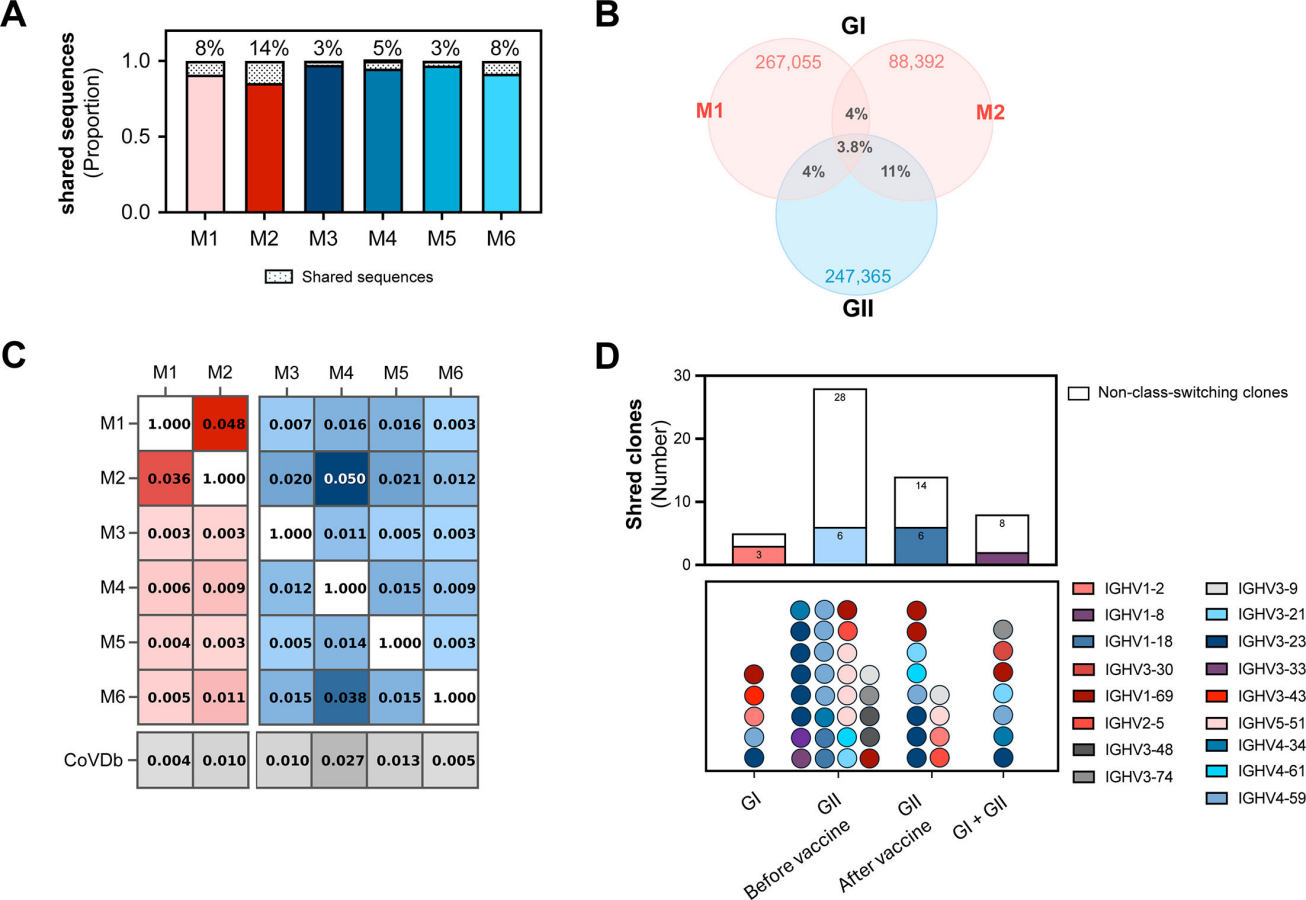
Convergence of B-cell clones between individuals and study groups. (**A**) Proportion of shared B-cell sequences compared to the total number of sequences in each individual. (**B**) Proportions of sequences shared between infected samples (group I) and between infected and vaccinated samples. (**C**) Heatmap illustrating the proportion of shared B-cell receptor sequences between pairs of samples across both groups. Each cell represents the pairwise comparison between two individual samples. Darker colors indicate a higher proportion of shared sequences. (**D**) Bar charts showing the number of shared clones classified based on class-switching properties (above chart) and based on V genes (lower chart). Each dot in the lower chart represents one clone.

Convergent clonotypes are important for unraveling viral epitopes that commonly induce antibody responses in multiple individuals. Here, we evaluated the presence and abundance of convergent clones among GI patients, GII individuals, and between the two groups (Fig. 6). Overall, GI shared 8% (M1) and 12% (M2) of their sequences with other individuals. The proportion of shared sequences in GII ranged from 3% in M3 and M5 to 8% in M6 (Fig. 6A). GI patients shared 4% of their sequences, while GII participants shared less than 1% of their sequences. M2 particularly shared 11% of sequences with GII participants (Fig. 6B). Pairwise analysis indicated the highest overlap between M1 and M2 (4%), M2 and M4 (5%), and M4 and M6 (3.8%). Comparison with the CoV-AbDab database (*n* = 12,536 sequences) showed minimal convergence, with shared clones making up only 0.4%–2.7% (mean = 1.2%, SD = 0.01) of total repertoires (Fig. 6C).

Five convergent clones were identified in GI, comprising 6,651 sequences. They belonged to IGHV3-43, IGHV1-69, IGHV3-23, and IGHV1-2. Notably, one of the shared IGHV3-43 clones appeared at multiple time points in both patients (abundance = 7.5% in M1 and 10.7% in M2), suggesting a potential role in the immune response. In GII, 34 convergent low-abundant clones were identified before vaccination, shared only between two individuals each. The majority (22/28) were non-class-switched and disappeared post-vaccination, indicating distinct, individual-specific responses to vaccination. Only eight convergent clones were found between groups, including two low-abundant IgG class-switched clones (Fig. 6D).

## DISCUSSION

MERS-CoV circulates in camels and causes severe sporadic outbreaks in humans, raising concerns about our preparedness for future outbreaks. Despite the extensive research, no vaccines or antiviral therapeutics are currently available. Besides, little is known about the dynamics of B-cell response during the acute phase of MERS-CoV infection. In this study, we characterized the B-cell response in two severe MERS-CoV cases with a history of SARS-CoV-2 exposure, as well as in MERS-CoV survivors both before and after SARS-CoV-2 vaccination.

The development of the immune response following MERS-CoV infection has been described in both acute and convalescent patients (24). In most cases, antibodies develop approximately 21 days after illness onset and can persist for 3–6 years after recovery (7, 8, 25–27). In our study, positive seroconversion was observed in both patients; however, the exact timing of seroconversion could not be determined due to the lack of samples from earlier time points. In M1 (a 50-year-old male), anti-MERS-CoV antibodies were detected on day 12, peaked at day 20, and remained elevated for the following 14 days. These high antibody titers were associated with a significant reduction in viral RNA levels in respiratory samples collected at later time points. In contrast, M2 (an 85-year-old male) exhibited high antibody levels on days 27 and 31 of illness; however, this was accompanied by persistent viral RNA shedding, a manifestation frequently observed in older MERS-CoV patients with underlying comorbidities (24, 28).

Cross-reactivity and cross-neutralization between MERS-CoV and SARS-CoV-2 are critical for understanding the role of MERS-CoV immunity in individuals with hybrid immunity (29). Cross-reactivity was clearly demonstrated in ELISA results, which showed that antibodies induced by MERS-CoV infection primarily targeted the S2 region of the SARS-CoV-2 spike protein, with low or undetectable reactivity against the RBD region. This was also evident in vaccinated individuals, as antibody titers against the full-length S and S2 proteins of both viruses showed positive correlations, further supporting enhanced cross-reactivity post-vaccination. These findings are consistent with studies reporting increased titers of cross-reactive antibodies following SARS-CoV-2 vaccination or infection in MERS-CoV survivors (17, 18, 30, 31).

Cross-neutralization activity was observed in both groups. High MERS-CoV neutralization levels in GI were accompanied by lower viral RNA levels. Both patients exhibited moderate-to-high neutralization activity (>50%) against SARS-CoV-2 despite relatively lower anti-SARS-CoV-2 antibodies, suggesting that this neutralization may have been mediated by cross-reactive MERS-CoV antibodies. Similar findings were reported in MERS-CoV survivors who experienced a boost in MERS-CoV neutralization following SARS-CoV-2 vaccination or infection without substantial changes in antibody titers (17, 18, 32). Similarly, vaccinated survivors exhibited a two- to fivefold increased MERS-CoV neutralization, despite the minimal increase in MERS-CoV antibody titers, suggesting the activation of vaccine-induced cross-reactive B cells. Collectively, these findings underscore the potential for identifying broadly neutralizing antibodies in patients with hybrid immunity.

Changes in serum antibody kinetics were associated with dynamic shifts in B-cell repertoire in GI. A dramatic increase in IgG and IgA class-switched clones was seen in M1, a profile commonly seen during infections (33, 34). This was accompanied by higher SHM frequency, clonal selection, and enhanced neutralization. The latter stage of infection was characterized by a decline in SHM frequency, stable neutralization levels, and virus clearance. This pattern reflects a transition from active germinal center reactions, which drive SHM and affinity maturation, to long-lived plasma cell production, a phenomenon also reported in other viral infections (35). Unfortunately, similar analyses could not be performed for M2 due to the lack of samples from earlier time points. However, samples collected 27 days post-infection demonstrated clonal expansion of mutated IgG clones accompanied by low diversity and high neutralization. This observation aligns with studies on severe COVID-19 cases, where extrafollicular responses dominate, leading to reduced repertoire diversity and expanded clones with high neutralization potential (36, 37).

GII, on the other hand, exhibited minimal alterations in B-cell repertoire following vaccination. They showed a slight decrease in diversity, an increase in IgG class-switched clones, and stable SHM frequencies. This suggests that vaccination mobilized antigen-experienced memory B cells without significantly altering overall repertoire properties, a pattern reported in studies on SARS-CoV-2 mRNA vaccines (38).

Analysis of IGHV genes revealed preferential usage of IGHV3-30, IGHV3-23, IGHV1-18, IGHV1-69, and IGHV4-59 genes in both groups, consistent with patterns observed in other viral infections (39–41). Group-specific analyses revealed distinct patterns of V gene usage. GI exhibited increased expansion of B-cell clones utilizing IGHV3-43 and IGHV4-34 genes that coincided with increased SHM frequency and enhanced neutralization. Both germlines were previously identified in MERS-CoV and SARS-CoV-2-neutralizing antibodies, highlighting their role in targeting conserved viral epitopes (39, 42). IGHV3-43 was also identified in pan-SARS-CoV-2-neutralizing antibodies (39, 42). In M2, the expansion of mutated IgG clones utilizing IGHV4-59 coincided with a marked increase in SARS-CoV-2 neutralization. IGHV4-59 clones were commonly reported following SARS-CoV-2 infection but not in MERS-CoV patients. Studies suggest that IGHV4-59 antibodies target conserved regions within the S2 subunit of SARS-CoV-2 spike protein, enabling cross-reactivity with other betacoronaviruses (43, 44).

In GII, distinct patterns emerged across individuals, with higher neutralization activity linked to the expansion of mutated IGHV4-34 clones in M5 and IGHV3-43 clones in M3 and M6. Additionally, the expansion of IGHV1-46, IGHV3-7, IGHV4-4, and IGHV3-53 clones was observed post-vaccination in some individuals. Of these, IGHV1-46 clones were identified following MERS-CoV and SARS-CoV-2 infections and in broadly neutralizing antibodies targeting the conserved S2 stem helix region of all three pathogenic coronaviruses (45, 46). These findings suggest that neutralization activity arises from a synergistic effort of multiple clones, with responses varying across individuals.

Mutation analysis revealed lower SHM frequencies in GI clones compared to GII, a pattern frequently seen in neutralizing antibodies during the acute viral infections (11, 47). Early immune responses prioritize rapid antibody production through extrafollicular B-cell activation rather than extensive affinity maturation in germinal centers (38). GII exhibited higher SHM frequencies, with no significant changes following vaccination. The higher baseline SHM levels in GII likely reflect the activation of memory B cells rather than *de novo* somatic hypermutation. Studies in vaccinated individuals reported robust B-cell responses and higher neutralization activity but no significant alteration in SHM (38, 48). These results highlight how infection and vaccination shape the B-cell repertoire. While infection induces a broader repertoire with lower SHM, vaccination primarily reactivates high-affinity memory B cells.

### Conclusion

In this study, we characterized B-cell kinetics in severe MERS-CoV-infected patients and survivors. Both groups showed enhanced neutralization activity against MERS-CoV and SARS-CoV-2. Specific IgG clones were linked to enhanced cross-neutralization. These findings demonstrate that individuals with hybrid immunity are a valuable source of potent, cross-neutralizing antibodies. However, there are several limitations in our study. First, we could not compare the immune response between the two cases at the early stage of infection, as the sample collection started after a month of symptom onset in the second case. Second, we did not test the antigenic specificity nor the neutralizing capacity for the identified clones. Instead, assumptions of their function were inferred from the association between clone expansion at a certain time point and neutralization activity at that point. To characterize the functionality of the clones, we compared our data with the CoV-AbDab database, which includes 71 MERS-CoV-specific antibody sequences. However, this approach is limited in breadth due to the limited number of MERS-CoV sequences in the database. Furthermore, the majority of SARS-CoV-2 sequences in the database are biased toward neutralizing RBD-specific antibody sequences. Therefore, future work should focus on isolating B-cell subsets that recognize the spike proteins of MERS-CoV, SARS-CoV-2, or both (cross-neutralizing) for further characterization.

## Data Availability

The raw sequencing data have been deposited to the National Center for Biotechnology Information (NCBI) database under accession no. PRJNA1261605. The code used to analyze sequencing data in the current study is available from the lead contact (Dr. Hebah Al-Khatib, h.alkhatib@qu.edu.qa) upon request.

## SUPPLEMENTAL MATERIAL

Supplemental material mbio.03356-25-s0001.pdf Supplemental table and figures.

ASM does not own the copyrights to Supplemental Material that may be linked to, or accessed through, an article. The authors have granted ASM a non-exclusive, world-wide license to publish the Supplemental Material files. Please contact the corresponding author directly for reuse.
