# Supplementary material for: Dynamics of B-cell response in MERS-CoV patients and survivors with hybrid immunity
Source: mBio. 2025 Dec 16;17(1):e03356-25. doi: 10.1128/mbio.03356-25 (PMC12802214; doi:10.1128/mbio.03356-25)
Supplement: Supplemental material — Supplemental table and figures. [file mbio.03356-25-s0001.pdf]

Supplementary Material

Supplementary Table 1: Timeline of MERS-CoV infection and SARS-CoV-2 vaccination for all subjects in both groups.

	MERS-CoV infection date				SARS-CoV-2 vaccination date*
**Sample**	**Timepoint 1**	**Timepoint 2**	**Timepoint 3**	**Timepoint 4**	
**M1**	21-Mar- 2022	23-Mar- 2022	28-Mar-2022	31-Mar-2022	Aug-2021
**M2**	07-Apr-2022	11-Apr-2022			Feb-2021
**M3**	Jun-2016	-	-	-	Jun-2021
**M4**	Aug-2017	-	-	-	Sep-2021
**M5**	Oct-2013	-	-	-	Dec-2021
**M6**	Oct-2013	-	-	-	Sep-2021

**All samples were collected after receiving the second dose of SARS-CoV-2 mRNA vaccine.*

Supplementary Figures

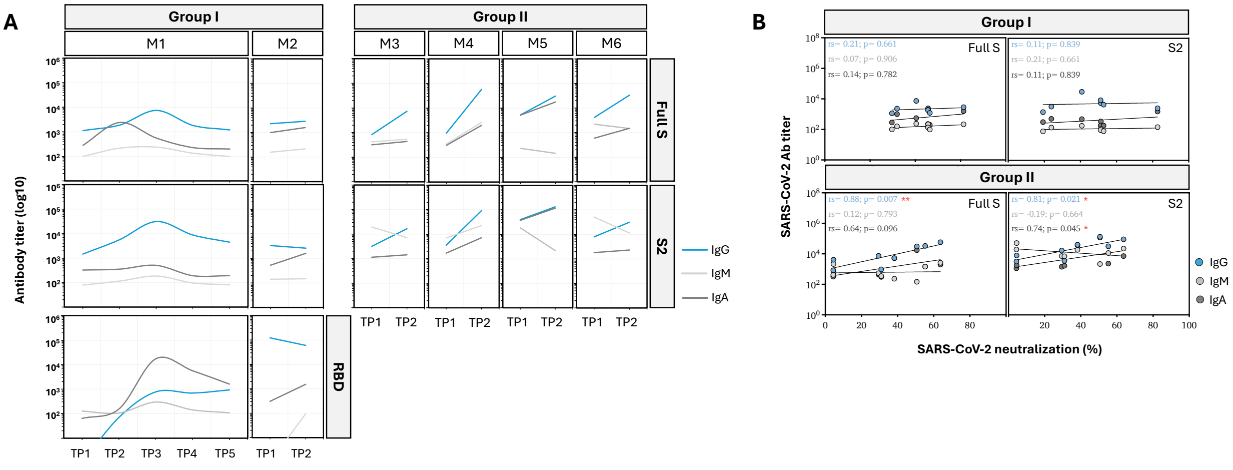

Supp. figure 1: SARS-CoV-2 antibody titers and their correlation with neutralization activity. (A) Antibody titers against SARS-CoV-2 proteins. The panel shows the titers of IgG, IgM, and IgA antibodies specific to SARS-CoV-2 spike protein (S) and its S2 subunit. Titers are presented as log-transformed values. (B) Correlation between SARS-CoV-2 neutralization and antibody titers. The figure displays Spearman correlation coefficients (rs) between virus neutralization activity and antibody titers for IgG, IgM, and IgA isotypes against S and S2 proteins. Red asterisks denote statistical significance: * $p < 0.05$, ** $p < 0.01$, *** $p < 0.001$.

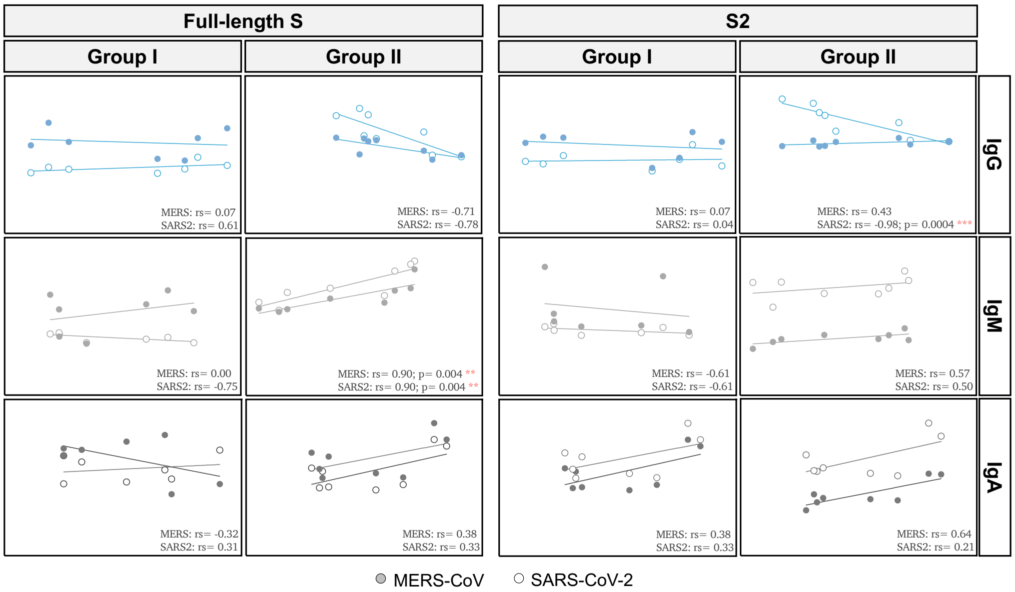

Supp. figure 2: Correlation between frequencies of each B cell isotype and corresponding serum antibody titers. Asterisks denote statistical significance: * $p < 0.05$, ** $p < 0.01$, *** $p < 0.001$.

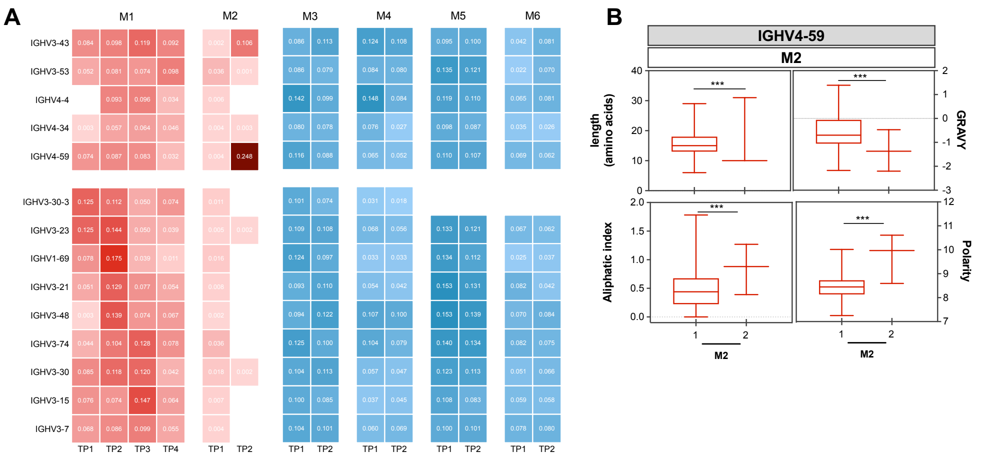

Supp. figure 3: Temporal dynamics of SHM frequency and physicochemical properties of specific B cell clones. (A) Temporal changes of SHM frequency in B cell clones across V gene families. **(B)** Changes in physicochemical properties of IGHV4-59 clones in patient M2.
